# Can acoustic structural quantification be used to characterize the ultrasound echotexture of the peripheral zone of breast lesions?

**DOI:** 10.3233/CH-180484

**Published:** 2019-08-05

**Authors:** Annika Bach, Clarissa Hameister, Torsten Slowinski, Ernst Michael Jung, Anke Thomas, Thomas Fischer

**Affiliations:** a Department of Radiology, University Berlin, Charité, Berlin, Germany; b Department of Nephrology, University Berlin, Charité, Berlin, Germany; c Department of Radiology and Interdisciplinary Ultrasound Centre, Universitary Hospital, Regensburg, Germany; d Department of Obstetrics and Gynecology and Ultrasound Research Laboratory, University Berlin, Charité, Berlin, Germany; e Department of Radiology and Interdisciplinary Ultrasound Centre and Ultrasound Research Laboratory, University Berlin, Charité, Berlin, Germany

**Keywords:** Ultrasound, breast neoplasms, breast cancer, breast lesion, B mode, ASQ, sonography, diagnostic imaging, halo, peripheral rim, peripheral zone, BI-RADS

## Abstract

**BACKGROUND:**

Besides mammography, breast ultrasound is the most important imaging modality for women with suspected breast cancer. New software tools bear high potential for improved detectability and specification of malignant breast lesions.

**OBJECTIVE:**

To compare the halo depicted around malignant breast lesions by ultrasound using Acoustic Structure Quantification (ASQ) of raw image data with the echogenic rim seen in B-mode ultrasound.

**METHODS:**

This retrospective study included 37 women for whom conventional B-mode ultrasound of the breast and ASQ were available as well as histopathology findings for comparison. Software tools were used to measure the halo area or echogenic rim and tumor area and calculate halo-to-lesion ratios for the two ultrasound modes. Six inexperienced readers characterized the breast lesions based on this information. Specificity, sensitivity, positive predictive value (PPV), and negative predictive value (NPV) were determined. ANOVA, the Wilcoxon test, and ROC curve analysis were performed.

**RESULTS:**

There was a linear relationship between ASQ-based and B-mode-based halo-to-lesion ratios; however, a systematic error was also noted. ASQ-derived ratios tended to be higher for breast lesions with lymphangioinvasion (*p* = 0.051, n.s.) and higher N-stages (*p* > 0.925, n.s.), while there was no correlation with other markers. Because of the significantly greater conspicuity of peritumoral halos in the ASQ mode, inexperienced readers achieved greater sensitivity (78% vs. 74%) and specificity (75% vs. 71%) and higher NPVs (75% vs. 71%) and PPVs (78% vs. 74%) compared with B-mode images. Greater halo conspicuity affected the identification of malignant lesions with both modes; ASQ was found to be particularly well suited (F_Bimage_ (1,100) = 19.253, *p* < 0.001; F_ASQ_ (1,100) = 52.338, *p* < 0.001). The inexperienced readers were significantly more confident about their diagnosis using the ASQ maps (z = –3.023, *p* = 0.003).

**CONCLUSIONS:**

We conclude that the halo in ASQ and the echogenic rim in B-mode ultrasound are attributable to different morphologic correlates. ASQ improves diagnostic accuracy and confidence of inexperienced examiners because of improved halo visibility.

## 1 Introduction

Besides mammography, breast ultrasound is the most important imaging modality for women with suspected breast cancer. Used as a supplement to mammography, ultrasound improves diagnostic sensitivity from 40–70% to up to 97% (depending on glandular density) [1]. An ultrasound examination improves both the detection of breast lesions and their further characterization [2, 3]. Conventional ultrasound criteria of malignancy include irregular margins, a long axis perpendicular to the skin, blurred contour, a heterogeneous echotexture, posterior acoustic shadowing, invasive growth, and increased perfusion [4, 5]. According to publications of the German Society for Ultrasound in Medicine (Deutsche Gesellschaft für Ultraschall in der Medizin, DEGUM), an echogenic rim surrounding an echolucent center is another sign of malignancy [6–8]. Such a rim was first described in the early 1990s [9] and is most likely attributable to tissue changes around a lesion due to displacement, tumoral invasion, microvascularisation [10–12] and/or or lymphangioinvasion. However, the underlying mechanism is still not fully understood.

Rapid advances in the development of medical ultrasound with the advent of new technical options such as compounding, tissue harmonic Imaging (THI), and speckle reduction have made B-mode ultrasound a more and more sensitive diagnostic tool, especially for the detection of minute tissue changes [3]. The use of contrast-enhanced ultrasound (CEUS) rises the diagnostic accuracy a lot and can help to differentiate between benign and malignant lesions [13–15]. On the other hand, the interpretation of different gray scales and perfusion patterns in ultrasound images is still highly subjective, and correct interpretation requires an experienced examiner. New, supplementary tools have been introduced to overcome these diagnostic limitations: primarily developed for the evaluation of liver cirrhosis, Acoustic Structure Quantification (ASQ; Toshiba Medical Systems, Otawara Japan) offers computer-based analysis of echo patterns based on B-mode images and computes deviations from the expected normal distribution according to the method of Rayleigh [16–18]. The deviation can be displayed in color. Applied to breast imaging, ASQ maps depict a halo of markedly different color around malignant breast lesions. This halo is similar to the echogenic rim known from B-mode ultrasound.

The aim of this study was to compare these two phenomena and establish possible correspondences. Another aim was to evaluate whether ASQ-based color displays can improve the differentiation of malignant and benign breast lesions when used by inexperienced examiners.

## 2 Material and methods

### 2.1 Patient selection and data

We retrospectively analyzed imaging findings and other data from a total of 37 women with histologically proven breast cancer. All breast lesions included in the analysis were detected in routine clinically examinations and were clearly visible sonographically (B-mode and ASQ). Histology (core biopsies or surgical specimens) was available for all focal breast lesions. Patients having undergone treatment before were not included. Written informed consent for study inclusion was available from all women. Data retrieved and analyzed were patient demographics, imaging results, histologic findings, and receptor status. The project was tested and approved (control no 731-16) according to §25 of the Berlin hospital law. All patients have signed a written informed consent.

### 2.2 Ultrasound

All patients included were examined in an ultrasound center associated with an interdisciplinary breast center using a high-end ultrasound machine (Aplio XG 500, Toshiba Medical Systems, Otawara, Japan) with a 5 cm linear broadband transducer. The examinations were performed at 9 to 14 Megahertz (MHz), depending on breast size and lesion depth, by a qualified radiologist with a level II certificate for breast ultrasound of the German Society for Ultrasound in Medicine (Deutsche Gesellschaft für Ultraschall in der Medizin, DEGUM), a DEGUM level III certificate in radiology, and several years of experience in breast ultrasound. Ultrasound images were optimized using spatial compounding, frequency-based compounding, *ApliPure*™ levels 5–7, *differential Tissue Harmonic Imaging (dTHI)*©, and *Precision Imaging*© with level 4-5 *Speckle Reduction* (*SR*). All focal breast lesions were documented in two planes (B-mode) perpendicular to each other with one plane showing the largest lesion diameter and information on lesion size and localization. Additionally, focal breast lesions were documented in other ultrasound modes (ASQ and elastography).

### 2.3 Acoustic Structure Quantification (ASQ) and parameters

**Fig.1:**
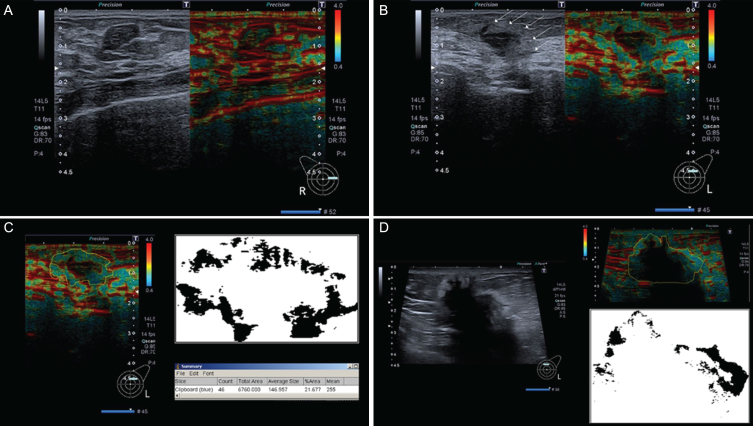
(A–D): Fibroadenoma without halo sign (A), small invasive breast cancer showing a clear surrounding halo (B), analysis of tumour in *B* using ImageJ (C), and a big, invasive breast cancer showing halo sign without an increased rim width in relation to halos of smaller breast cancers like in A (D).

Acoustic Structure Quantification (Toshiba Medical Systems, Otawara, Japan) is a new B-mode-based software tool that was originally developed for evaluation of liver cirrhosis/fibrosis. Details of the underlying physical and statistical principles have been published before [17–19]. Briefly, raw ultrasound data are used to obtain both qualitative (color-coded parameter map) and quantitative information (echo amplitude analysis, deviation from estimated normal values) on the tissue of interest. For this purpose, the examiner places a primary region of interest (ROI) in the target tissue, and the software generates several secondary ROIs within the primary ROI. Reflected echo amplitudes in the ROIs are used to estimate a normal value (Rayleigh distribution), and ASQ measures the deviation of measured values from the predicted normal value. The results are Cm^2^ values, which can be displayed in histogram form or as a parametric color map. ASQ-based parametric maps show a blue perifocal zone (halo) around malignant breast lesions, which is similar to the echogenic rim known from B-mode images. Benign lesions do not have a halo. The Java-based image processing software ImageJ (version 1.48) was used to measure halo or rim and tumor areas of breast lesions in ASQ maps and B-mode images (Fig. 1).

### 2.4 Diagnostic performance of inexperienced examiners

To determine whether the halo seen in ASQ maps, which corresponds to the echogenic rim surrounding malignant breast lesions in B-mode images, facilitates the diagnosis for inexperienced examiners, six advanced-level medical students interpreted the images. Before reading the images, the students received a brief introduction to the BI-RADS classification of breast lesions, malignancy criteria, and the ASQ technique. In addition, they were shown examples of B-mode images and ASQ maps of one malignant and one benign breast lesion. During the reading, the students were presented 17 images (nine malignant and eight benign lesions) without additional clinical data. They assessed images using a questionnaire asking them to assign a BI-RADS category, assess halo conspicuity on a 4-point rating scale (0 to 3), and rate their diagnostic confidence on a scale of 0 to 10. The students gave written informed consent to analysis and publication of the results.

### 2.5 Statistical analysis

For statistical analysis, ratios of the area of the perifocal zone (halo or rim) and of the lesion were calculated for ASQ maps and B-mode images and will be referred to as halo-to-lesion ratios for both ultrasound modes in the following. The halo-to-lesion ratios are displayed in a Bland-Altman diagram. Pearson’s linear regression was used to analyze the relationship of halo-to-lesion ratios between ASQ and B-mode ultrasound. Analysis of variance (ANOVA) was performed to correlate halo-to-lesion ratios with the prognostic factors analyzed. For one prognostic factor - lymphangioinvasion — we additionally calculated the area under the curve (AUC) and the receiver operating characteristic curve (ROC) to validate the prognostic role of the halo-to-lesion ratio. Diagnostic performance of the inexperienced examiners was analyzed to determine specificity and sensitivity for the differentiation of benign and malignant breast lesions in B-mode images and ASQ maps as well as positive and negative predictive values. B-mode and ASQ images were compared in terms of diagnostic confidence and halo conspicuity.

IBM SPSS Statistics for Microsoft Windows, version 19.0.0.1, and IBM SPSS Statistics for Macintosh, version 23.0, supplemented by Microsoft^®^ Excel^®^ 2008 for Macintosh, version 12.3.6, were used for descriptive and statistical analysis. A *p*-value <0.05 was considered to indicate statistically significant differences.

## 3 Results

### 3.1 Patient characteristics/study population

The 37 women included in the study had an average age of 59±15 years. TNM stages and histologic grades were available for 24 patients (Table 1) — 13 women had undergone core biopsy. In these 24 patients, histologic grading identified 2 G1 tumors, 15 G2 tumors, and 7 G3 tumors. Lymphangioinvasion was identified in 5 women. Overall, 78% of patients (*n* = 29) had a histopathologic carcinoma of no special type (NST), 19% (*n* = 7) invasive lobular carcinoma (ILC), and 3% (*n* = 1) other subtypes. Immunohistochemically, 22 patients had low HER2/neu expression while 4 patients each had moderate and high expression. No HER2/neu expression was detectable in 2 women, and expression was not determined in 5 patients. The distribution of proliferation indices is presented in (Table 1). Estrogen receptor (ER) status was positive in 27 patients (100% in 20, 90% in 5, and 80% in 2 patients) and negative in 10 patients. A total of 23 patients had progesterone-receptor (PR)-positive tumor cells (100% in 7, 80% in 4, 60% in 3, and <50% in 9); 14 patients were PR-negative. Histologic sum scores were available for 35 patients (95%) and ranged from 4 to 9 (see Table 1). Maximum lesion diameters measured in surgical specimens were available for 24 patients. Diameters ranged from 9 mm to 68 mm with a mean of 23.1±14 mm. Sum scores were 4 in 2 patients, 6 in 16 patients, 7 in 3 patients, 8 in 10 patients, and 9 in 4 patients (Table 1).

**Table 1:** Patient characteristics

	No.	Percentage		No.	Percentage
Total population	37	100%		37	100%
Age, median (SD), years	59±14,5		Her2-neu expression
Age distribution, years			low	22	59%
18–30	0	0%	moderate	4	11%
31–45	7	19%	high	4	11%
46–60	12	32%	none	2	5%
>60	18	49%	not available	5	14%
Type of breath cancer			Proliferaion index Mib-1
NST	29	78%	up to 25%	28	76%
ILC	7	19%	25–50%	4	11%
Other	1	3%	50–75%	2	5%
T-Stage – tumor size			over 75%	2	5%
T1	14	38%	not available	1	3%
T2	9	24%	Hormone receptor status
T3	1	3%	ER status	27	73%
T stage not available	13	35%	PR status	23	62%
N-Stage – nodal metastasis			Histologic sum score
N0	12	32%	4	2	5%
N1	4	11%	5	0	0%
N2	3	8%	6	16	43%
N3	1	3%	7	3	8%
N stage not available	13	35%	8	10	27%
Grading			9	4	11%
G1	2	5%	Tumor diameter (surgical specimen)
G2	15	41%	up to 2 cm	14	38%
G3	7	19%	over 2 cm	8	22%
Not available	13	35%	over 4 cm	2	5%
Lymphangioinvasion	5	14%

### 3.2 Halo-to-lesion ratios in ASQ and B-mode ultrasound

**Fig.2:**
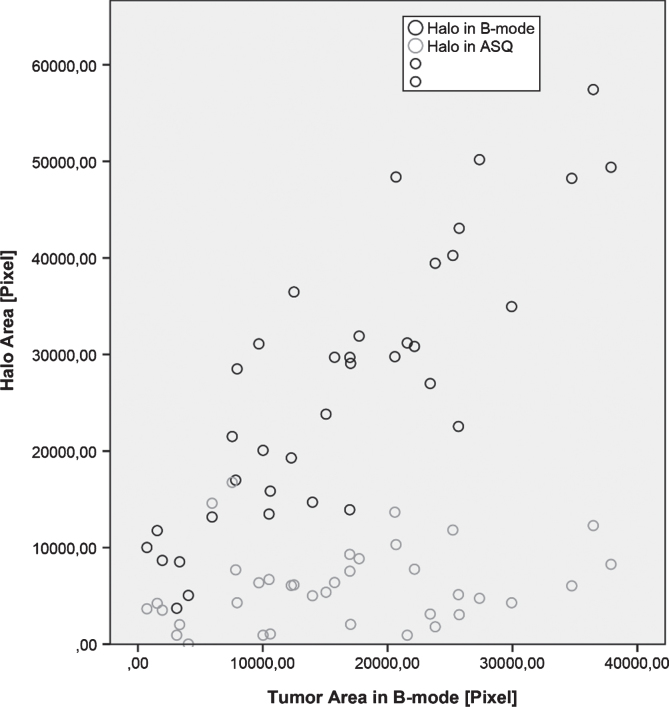
Distribution of halo-to-lesion ratios derived from B-mode images and ASQ maps.

**Fig.3:**
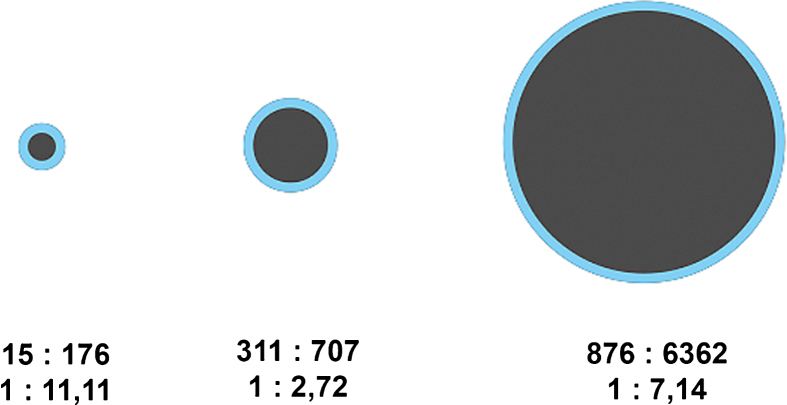
Diagram of halo-to-lesion ratios derived from ASQ maps for different tumor sizes – the halo does not increase in proportion to tumor size but appears to have a fairly constant width.

For each breast lesion, the tumor area and the area of the echogenic rim or halo were measured to calculate the halo-to-lesion ratio. While there was a positive linear correlation between tumor areas in ASQ and B-mode images (R^2^ = 0.682), the ASQ-based tumor area was consistently larger and the halo area consistently smaller compared with B-mode images. Therefore, with the exception of two cases, the halo-to-lesion ratio was <1 in the ASQ mode and >1 in the B-mode. There was a positive linear relationship between lesion and halo areas, which was strong in the B-mode and weak in the ASQ mode (R^2^ = 0.733 versus 0.202). The ASQ-based halo area was thus much less dependent on breast cancer size than the echogenic rim in B-mode images. When related to the tumor area measured in B-mode images, the ASQ-based halo area was found to increase with tumor size while it was nearly constant in relation to the tumor area measured ASQ maps (Figs. 2 and 3).

The comparison of halo-to-lesion ratios determined with the two ultrasound modes revealed no interdependence. Hence, size ratios differ between the ASQ and B-mode. These results suggest that the halo identified with the ASQ technique depends on a different underlying mechanism than the echogenic rim seen in conventional B-mode ultrasound.

### 3.3 Correlation of halo-to-lesion ratio with histology

**Fig.4:**
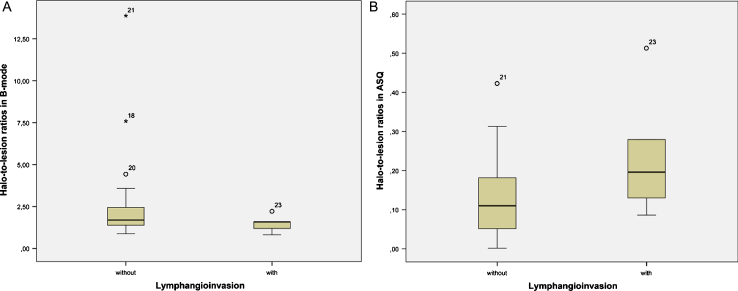
Distribution of halo-to-lesion ratios for tumors with and without lymphangioinvasion in B-mode images (A) and ASQ maps (B).

**Fig.5:**
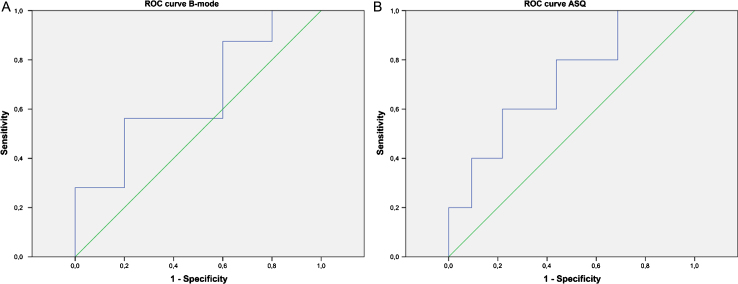
ROC curves of halo-to-lesion ratios derived from B-mode images (A) and ASQ maps (B) for predicting lymphangioinvasion of malignant breast lesions.

**Fig.6:**
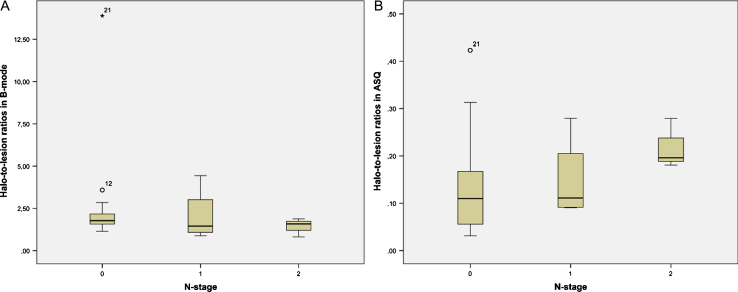
Distribution of halo-to-lesion ratios for N-stages 0 to 2 derived from B-mode images (A) and ASQ maps (B).

Halo-to-lesion ratios in ASQ maps and B-mode ultrasound were compared with different prognostic markers. Interesting observations were made for N-stage and lymphangioinvasion (Table 2). The ASQ-based halo-to-lesion ratio tended to increase when lymphangioinvasion was present. A similar observation was not made for the B-mode-based halo-to-lesion ratio (Fig. 4). ROC curve analysis also confirmed a higher discriminatory power in predicting lymphangioinvasion for the ASQ-based halo-to-lesion ratio compared with the ratio determined from B-mode imaging (*p* = 0.131 for ASQ vs *p* = 0.267 for B-mode) (Fig. 5). Hence there was a trend toward a positive relationship between lymphangioinvasion and an increase in the halo area relative to the lesion area (i.e., increasing halo-to-lesion ratio) for the ASQ mode. In addition, the ASQ-based halo-to-lesion ratio tended to increase with higher N-stages. While the distribution was similar for N0 and N1, the difference between N1 and N2 lesions was visually apparent although it did not reach statistical significance (N0: *M* = 0.15, *SD* = 0.03; N1: *M* = 0.15, *SD* = 0.04; N2: *M* = 0.22, *SD* = 0.03). No such trend was observed for the ratios derived from B-mode ultrasound (Fig. 6).

**Table 2:** Correlation of halo-to-lesion ratios derived from ASQ maps and B-mode images with different prognostic markers including Pearson correlation coefficients

	Halo-to-lesion ratio in B-mode	N-stage	Lymphangio-invasion
Halo-to-lesion ratio in ASQ	0.528^*^	–0.017	0.323^**^
Halo-to-lesion ratio in B-mode		–0.092	–0.145

Pearson correlation coefficient. ^*^Correlation significant at 0.01 (two-tailed). ^**^Two-tailed significance of correlation at *p* = 0.051.

### 3.4 Diagnostic confidence of inexperienced examiners using B-mode and ASQ

For comparison of B-mode ultrasound and ASQ maps of breast lesions, six readers assessed both modalities and made a diagnosis.

There was moderate agreement between B-mode images and ASQ maps in differentiating benign and malignant breast lesions (*κ*= 0.449, *p* = 0.000), with a larger number of true positive results when readers used the ASQ maps. This resulted in an overall higher diagnostic accuracy for ASQ with 78% sensitivity and 75% specificity compared with slightly lower results of 74% and 71%, respectively, for B-mode imaging.

Conspicuity of the halo (ASQ) and echogenic rim (B-mode) of malignant breast lesions was assessed on a 4-point scale from 0 (nonvisibility) to 3 (excellent visibility). Mean conspicuity scores were 1.63 (SD 0.784) for B-mode images versus 2.20 (SD 0.833) for ASQ. Halo conspicuity on ASQ maps was found to be superior to B-mode ultrasound in 46 cases (45%), to be the same in 36 cases (35%), and to be inferior in 20 cases (20%). In terms of lesion characterization, there was a significant difference in halo conspicuity for malignant lesions between B-mode and ASQ maps (*U* (54, 54) = 900, *p* = 0.000) but not for benign lesions (*U* (48, 48) = 1150, *p* = 0.988). The students reported greater diagnostic confidence using ASQ maps compared with B-mode ultrasound. Overall, diagnostic confidence was rated superior for ASQ in 60 cases, the same for both modes in 30 cases, and inferior to B-mode in only 12 cases (*p* < 0.001). Halo conspicuity in the two modes appeared to have a significant positive effect on diagnostic confidence (B-mode: *AUC* = 0.728, *SD* = 0.051, *p* = 0.000; ASQ: *AUC* = 0.826, *SD* = 0.041, *p* = 0.000). Diagnostic confidence increased with halo conspicuity, and ASQ maps were superior in terms of the resulting diagnostic accuracy.

## 4 Discussion

Breast ultrasound is the second most important imaging modality following mammography for the detection and characterization of suspicious breast lesions, especially in women with dense glandular tissue and when mammography is inconclusive [1, 20]. Since the interpretation of breast ultrasound findings was not standardized in the past, the American College of Radiology (ACR) proposed a BI-RADS classification for ultrasound based on the well-established BI-RADS classification for mammography. Ultrasound criteria for breast lesion characterization include long axis orientation, shape, echotexture, margins, echogenicity posterior to the lesion, appearance of surrounding tissue, presence of calcifications, and lesion vascularization. Since its introduction, this BI-RADS classification has been used for standardized reporting of breast ultrasound findings and quality assurance and as a standard in scientific studies [21, 22]. An echogenic rim around malignant breast lesions was described as early as the 1990s and is mentioned as a criterion in the first 4 editions of the BI-RADS classification [8, 9, 21]. This rim is no longer mentioned in the 5th edition of the ultrasound BI-RADS classification but is still listed as a malignancy feature in the DEGUM recommendation, which is analogous to the BI-RADS classification [23].

Our results confirm that the echogenic rim in B-mode images and the halo in ASQ maps are valid criteria of malignancy. Contrary to initial assumptions, however, the rim and the halo appear to be attributable to different underlying pathophysiologic mechanisms. The two show distinct behavior in relation to lesion size: while the rim area increases with the lesion area, the halo in ASQ maps appears to be fairly constant and shows little dependence on lesion size (Figs. 1–3). Hence, our results suggest that, while both ultrasound phenomena are associated with malignant breast lesions, they are caused by different mechanisms. It is most likely that the ASQ-based halo is a component of the rim seen in B-mode ultrasound. Alternatively, the rim overlays the halo.

The only well-established peripheral feature of malignant lesions known so far that is fairly constant regardless of lesion size is peritumoral edema. It has been described for many tumors in different organs (Baltzer, 12–14, 30, 35). Perifocal edema is also listed among the malignancy criteria in the ACR BI-RADS catalogue (Baltzer 8, 17). Several recent studies confirm a significantly higher prevalence of peritumoral edema in patients with malignant breast lesions versus those with benign lesions [24–28]. These findings were obtained with high-resolution T2-weighted MRI sequences, which show peritumoral edema as a bright rim around malignant lesions. Here, perifocal edema is the most common and strongest malignancy feature, comparable to diffuse unilateral or bilateral edema [25, 26]. Baltzer et al. report peritumoral edema to be highly specific for malignant breast lesions, observing an association with higher tumor grades and larger tumor size [25]. They suggest that the absence of edema around smaller tumors may be due to limited image resolution. Baltzer et al. also propose a malignancy score for MRI findings, which includes peritumoral edema as one of five malignancy criteria, and report an accuracy of over 95% for this score [24]. Their observations on edema in MRI are consistent with our results on the halo seen in ASQ maps, which also tends to be associated with higher N-stages and a larger tumor area. At the same time, like perifocal edema, the halo width is fairly constant regardless of tumor size. Dietzel et al. reported a strong positive association of peritumoral edema with the presence of axillary lymph node metastasis [26]. This is also consistent with our finding of larger halo-to-lesion ratios when lymphangioinvasion was present or patients had higher N-stages. Note, though, that here we only found trends without statistically significant differences; however, this is most likely attributable to the small study population of only 37 patients. Our assumption that the ASQ-based halo reflects edema is mainly based on its perifocal location, size correlations, and its association with malignant breast lesions. A further evaluation by contrast-enhanced MRI could help to prove or disprove our assumption by visualizing microvascularisation and/or edema around the lesions.

However, it should be kept in mind that, while benign breast lesions are reported to have no halo in B-mode ultrasound, a halo can sometimes be seen around intraductal papillomas, radiation scars and/or lobular tumors. Also lymph nodes can show an echogenic rim. These phenomena may be caused by different pathomechanisms. In case of doubt about the lesion’s malignancy or benignity, it should also be further evaluated by contrast-enhanced MRI and/or CEUS [13, 14]. Especially contrast-enhanced ultrasound (CEUS) with perfusion analysis offers new possibilities for the dynamic evaluation of microvascularitsation in carcinomas and benign lesions [15, 29].

The second focus of our study was on the characterization of breast lesions by inexperienced examiners. This is highly relevant given that ultrasound is the imaging modality that most strongly relies on examiner experience in terms of sensitivity and specificity. An intuitive and structured sonographic approach is especially helpful in centers with a large proportion of residents or radiologists beginning to use ultrasound in a new area. Such an approach helps reduce misinterpretation. Our results clearly show that visualization of the halo in ASQ maps clearly improves lesion characterization by less experienced readers. While better halo conspicuity affected the diagnosis of malignant breast lesions for both ultrasound modes (each *p* < 0.001), conspicuity was significantly greater in ASQ maps compared with B-mode images. Consistently, the ASQ maps had greater sensitivity (78% vs. 74%) and specificity (75% vs. 71%) and higher NPVs and PPVs (75% vs. 71% and 78% vs. 74%, respectively) compared with B-mode ultrasound when interpreted by inexperienced examiners. Also, the inexperienced examiners reported significantly better subjective diagnostic confidence in interpreting the ASQ maps (*p* = 0.003). As we are not aware of any similar study including inexperienced readers, we cannot compare our findings with published data. However, our initial results are encouraging and suggest that ASQ is a promising tool for improving the sonographic assessment of breast lesions in the future. Wang has shown recently that using three-dimensional (3D) shear wave elastography (SWE) combined with BI-RADS is more beneficial compared to BI-RADS alone. Furthermore 3D-SWE reduces inter-rater reliability. ASQ or an adapted method could similarly contribute to predicting breast cancer and reduce interobserver variability [30].

An important advantage and novel aspect of the ASQ mode is the option of generating parametric maps. A disadvantage of the ASQ mode, which was originally developed for liver ultrasound, is that statistical processing of the raw data assumes a Rayleight distribution for normal tissue [17]. While the scatter radiation in breast tissue approximates Rayleigh distribution, other models such as K-distribution [31, 32] or Nakagami distribution [33] may better describe the behavior of ultrasound in breast tissue. Using non-Rayleigh-based statistical methods in conjunction with ultrasound for the diagnostic assessment of breast lesions was first proposed by Shankar et al. as early as 1993 [34]. The benefit of using statistical methods for image processing was also confirmed by Ito et al. in 2005 [35]. We therefore conclude that adjustment of the algorithms used for ASQ to the characteristics of breast tissue, e.g., by assuming K-distribution, would further improve ultrasound diagnosis using this technique.

Our study is limited by its retrospective design and a small study population of only 37 patients. Testing the method with only six inexperienced readers also limits is usefulness in terms of obtaining statistically meaningful results. It is therefore desirable to further investigate ASQ in larger, prospective studies of women with breast lesions. To correlate the halo depicted by ASQ with perifocal edema, comparative studies are warranted that use an imaging modality clearly visualizing peritumoral edema. Currently, only MRI provides sufficient resolution for this purpose and should therefore be used in future studies investigating ASQ and the halo sign of breast lesions.
